# Electrochemotherapy of head and neck cancer in elderly patients: a preliminary report

**DOI:** 10.1186/1471-2482-13-S1-A5

**Published:** 2013-09-16

**Authors:** R Benevento, A Vicidomini, V Padovano Sorrentino, M Renzulli, D Di Nardo, S Canonico, A Santoriello

**Affiliations:** 1 Department of Medical, Surgical, Neurologic, Metabolic and Ageing Sciences, Second University of Naples, Naples, Italy; 2 Department of Head and Neck Pathology, Second University of Naples, Naples, Italy

## Background

Locoregional recurrence is the most common cause of failure after head and neck cancer surgery and results in significant morbidity including effects on speech and swallowing and on cosmetic disfigurement [1].

Increasing age itself is an important risk factor for surgical treatment, and older people have coexisting medical conditions that can adversely affect surgical care and outcomes, and these must be taken into consideration. When surgical excision is not possible there are several therapeutic options.[2] Electrochemotherapy (ECT) is defined as a local treatment which, via cell membrane permeabilising electric pulses, potentiates the cytotoxicity of non permeant or poorly permeant anticancer drugs with high intrinsic cytotoxicity at the site of electric pulse application. Electroporation transiently permeabilizes tumor cell membranes, thus enabling diffusion of a chemotherapeutic drug into the cells and increasing its cytotoxicity. The most frequently used drugs are bleomycin and cisplatin. Association with electroporation induces a cytotoxicity increase of up to 80-fold for cisplatin and up to 8000-fold for bleomycin.

Intravenous bleomycin is administered at the dose of 15.000 IU/m2, in bolus.

ECT has significantly higher effectiveness than chemotherapy alone. [3]

## Methods

This prospective observational study took place throughout July 2011 to March 2013.

Eight consecutive elderly patients (6 men and 2 women, median age of 81 years) with secondary, recurrent or metastatic tumours of the head and neck or skin, for which either no curative treatment options were available, were treated with ECT . Histologically confirmed squamous cell carcinomas of the head and neck area in six, and tumors of salivary glands in two patient were eligible for treatment. The tumor was measured. The maximum diameter of the tumour should be 8.0 cm and the upper limit of volume to be treated was 80.0 cm3 for head and neck tumours. Inclusion criteria were as follows: the tumors were unsuitable for resection or radiotherapy; they were unresponsive to, or progressed after, two or three lines of systemic chemotherapy; the patient Karnofsky performance status was greater than 70%. Patient enrollment was carried out according to the ESOPE criteria.

Exclusion criteria were: coagulation disorders, allergic reaction to bleomycin, epilepsy, chronic renal dysfunction (creatinine ≥ 150 lmol/L), patients with clinically manifeste arrhythmia or pacemaker.

Bleomycin administration was followed by the application of brief electric pulses to each tumor nodule within 8 min after intravenous infusion of the drug .

All patients were treated under general anesthesia.

The patients were followed up after 8 weeks by clinical examinations and 3, 6, months after treatment.

## Results

The procedure was well-tolerated by all patients. No ECT-related serious adverse events were reported. The procedure lasted a median of 25 min (range 15–35) and the median hospital stay was 1 day (1-3day) A total of 10 different lesions have been treated in these patients. Local tumour control was reached in 9 of the 10 (90 %) tumours.

Of the 10 lesions assessable for the study, 5 (50%) showed a complete response, 3 (30%) a partial response, 1 (10%) stable disease and 1 (10%) progression of the disease. The objective response 3 months after the procedure was 80%. After a follow-up of 2 to 12 months, 25% of the patients were alive and free of disease, 50% were alive with disease, 12.5% died for disease and 12.5% died for other cause.

We observed cytoreduction of large lesions (92%), bleeding control (100%), reduction of seroma ( 90%), and absence of painful(100%) . No infections were detected clinically.

Seven patients received a single ECT session (87.5%). A second treatment was performed in 1 patients (12.5%) after 3 months after the first treatment.

## Conclusion

Increasing age itself is an important risk factor for surgical candidates. Surgical morbidity exhibited a linear increase across all age groups.

Several clinical reviews have reported on effectiveness of ECT. Electrochemotherapy can be proposed as alternative treatment modality to conventional therapies [2,3]

ECT is still considered a palliative treatment that should be reserved for patients with an advanced stage of disease and therefore not candidate for conventional cancer treatments.

ECT is a feasible alternative in case of inoperable tumors, located in pre-irradiated areas and resistant to chemotherapy [3]

In the last years, ECT has been proposed as a novel therapeutic weapon for the control of cutaneous, subcutaneous or mucosal neoplastic lesions. In the literature, few data exist about the effectiveness of the procedure for the treatment of head and neck tumors, but the reported objective response rates seem promising, ranging from 56% to 100%. [1] Our data seem to be comparable to those of the literature; in fact, we had an objective response rate of 80%.

The absence of systemic side effects and the low impact on the immune system also make this treatment suitable for elderly people and patients with poor physical condition even with repeated courses.
